# Systemic lupus erythematosus and atherosclerosis: immune pathways and the uncharted territory of gut microbiota and metabolism

**DOI:** 10.3389/fimmu.2025.1492726

**Published:** 2025-04-28

**Authors:** Quanren Pan, Xuemei Huang, Chaobin Liu, Qingjun Pan, Shian Huang

**Affiliations:** ^1^ Guangdong Provincial Key Laboratory of Autophagy and Major Chronic Non-communicable Diseases, Clinical Research and Experimental Center, Department of Nephrology, Affiliated Hospital of Guangdong Medical University, Zhanjiang, China; ^2^ Laboratory of Cardiovascular Diseases, Cardiovascular Medicine Center, Affiliated Hospital of Guangdong Medical University, Zhanjiang, China; ^3^ Department of Clinical Laboratory, State Key Laboratory of Respiratory Disease, The First Affiliated Hospital of Guangzhou Medical University, Guangzhou, China

**Keywords:** systemic lupus erythematosus, atherosclerosis, immune dysregulation, gut microbiome imbalance, metabolic disorders

## Abstract

Patients with Systemic Lupus Erythematosus (SLE) are significantly more susceptible to atherosclerosis, which may elevate their mortality risk. The review explores recent understandings of the origins and remedies for atherosclerosis associated with SLE. Our focus is particularly on the consequences of immune system disparities, interruptions in intestinal bacteria, and metabolic complications. The influence of SLE on atherosclerosis extends past usual risk elements, including processes specific to the disease. The list encompasses excessive immune cell activity, production of autoantibodies, inflammatory responses. A variety of therapies for atherosclerosis linked to SLE encompass cholesterol-lowering medications, anti-inflammatory drugs, immune suppressors, antimalarials, interferon treatments, NET inhibitors, and methods aimed at T and B-cells. However, existing research has its shortcomings, necessitating additional clinical trials to ascertain the efficacy and security of these therapies. The direct interactions among SLE, gut microbiota, metabolism, and atherosclerosis is underexplored, presenting innovation opportunities. Research into specific gut microbial strains and metabolites’ effects on immune responses and atherosclerosis progression in SLE patients is needed. Such research could uncover novel therapeutic targets and biomarkers, advancing prevention and treatment strategies for SLE cardiovascular complications.

## 1 Introduction

Patients with systemic lupus erythematosus (SLE) face an elevated risk of cardiovascular diseases (CVDs), particularly atherosclerosis (AS), driven by both traditional and SLE-specific factors. Traditional risk factors such as dyslipidemia, hypertension, and smoking interact with SLE-related pathological mechanisms, including chronic inflammation, immune dysregulation (1, 2).

Macrophages contribute to vascular injury through oxidized low-density lipoprotein (ox-LDL) uptake and foam cell formation (3). Type I interferons (IFNs) enhance neutrophil extracellular traps (NETs) release, exacerbating endothelial dysfunction (4, 5). CXCR3^+^ T helper 1 (Th1) cells migrate to arterial walls, promoting vascular inflammation, while B cells drive atherosclerosis via autoantibody production (e.g., anti-endothelial cell antibodies) and immune complex deposition (6–8).

Pro-inflammatory gut microbiota elevate systemic inflammation (9, 10), and dietary L-carnitine conversion to trimethylamine N-oxide (TMAO) by *Emergencia timonensis* accelerates atherosclerosis in mice and humans (11). SLE patients exhibit gut dysbiosis characterized by reduced beneficial bacteria and increased pathogenic species (12, 13). However, direct evidence linking gut microbiota or metabolites to SLE-associated atherosclerosis remains sparse, highlighting the need for further research.

We have focused on discussing immune-targeted therapies (e.g., interferon inhibitors (14), T-cell (15), and B-cell therapies (16)) and inflammatory pathways. Emerging approaches modulating gut microbiota or metabolites (e.g., TMAO inhibitors) show preclinical potential to attenuate plaque inflammation, though clinical validation in SLE remains scarce (17). This underexplored axis offers novel therapeutic opportunities.

This review aims to offer novel insights and new research directions for atherosclerosis associated with SLE. Current therapeutic strategies for SLE-related atherosclerosis prioritize immune balance restoration (e.g., interferon inhibition and T/B-cell modulation). Simultaneously, targeting gut microbiota dysbiosis and metabolites like TMAO emerge as promising adjunct approaches to ameliorate both autoimmune dysregulation and atherosclerosis in SLE. Integrating these dual pathways may pioneer synergistic therapies to reduce cardiovascular burden in SLE patients.

## 2 The role of immune imbalance in the pathogenesis of SLE-related atherosclerosis

### 2.1 Pro-inflammatory environment, oxidative stress, and macrophage dysfunction

**Figure 1:**
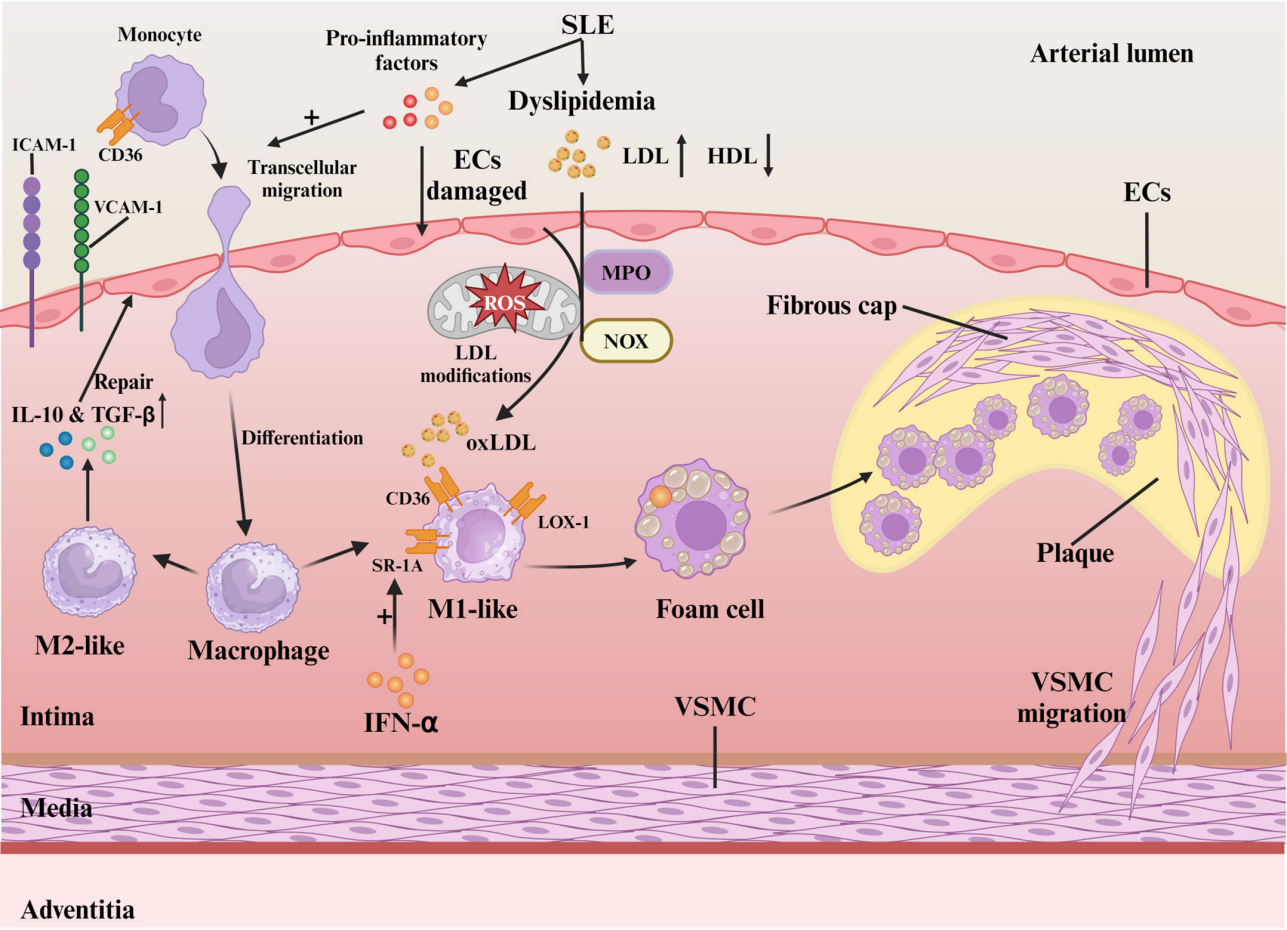
Mechanisms of oxidative stress and macrophage in SLE-associated atherosclerosis. Activated pro-inflammatory cytokines in SLE blood induce endothelial cell damage and monocyte migration via ICAM-1/VCAM-1. Differentiated macrophages polarize into M1/M2 based on local cues. LDL oxidation by myeloperoxidase and NADPH oxidase forms oxLDL, which M1 macrophages internalize via CD36, SR-A1, and LOX-1, leading to foam cell formation and plaque progression. VSMCs reinforce plaque stability by forming a fibrous cap.

The pro-inflammatory environment, oxidative stress, and macrophage dysfunction constitute key mechanisms driving SLE-related atherosclerosis (**Figure 1**). SLE, characterized by chronic inflammation, induces endothelial cell damage and dysfunction (18). Pro-inflammatory cytokines (e.g., TNF-α, IFN-γ) upregulate endothelial adhesion molecules, facilitating monocyte-laden leukocyte migration into the vascular intima (19). Patients with SLE exhibit a distinct “lupus lipoprotein pattern” with reduced high-density lipoprotein (HDL) and elevated low-density lipoprotein (LDL), total cholesterol, and triglycerides (20). This lipid profile synergizes with chronic inflammation to promote vascular injury and plaque initiation (21). Oxidative stress, defined by reactive oxygen species (ROS) overproduction, critically accelerates AS pathogenesis (22). ROS increase vascular permeability (23), and convert LDL to oxidized LDL (oxLDL) that accumulates subendothelially (3). Key enzymes like myeloperoxidase (MPO) and NADPH oxidase (NOX) are involved in this oxidative process. Oxidized LDL (oxLDL) is particularly critical in atherosclerosis, inducing inflammation through scavenger and Toll-like receptors (TLR) (24). OxLDL promotes the migration of monocytes towards the vascular endothelium and facilitating monocyte chemotaxis and adhesion through interactions between surface molecules like CD36 on monocytes and endothelial adhesion molecules such as vascular Cell Adhesion Molecule-1 (VCAM-1) and Intercellular Adhesion Molecule-1 (ICAM-1) (3).

Macrophage SRs (CD36, SR-A1, LOX-1) mediate oxLDL uptake, forming foam cells essential for AS progression (3, 25). M1 macrophages with high CD36/SR-A1 expression dominate early lesions (26), whereas M2 counterparts with reduced LOX-1 and elevated IL-10/TGF-β secretion exert protective effects (26). In SLE, IFN-α-induced SR-A1 overexpression (27) combined with elevated LDL (28) exacerbates foam cell formation. Patient-specific SR expression patterns modulate oxLDL clearance efficiency and inflammatory responses (25). Macrophage-T cell crosstalk in plaques sustains cytokine-driven inflammation (26). Polarization dynamics determine plaque fate: M1-derived pro-inflammatory mediators destabilize plaques (29), while M2 macrophages promote stabilization (29). oxLDL-driven M1 polarization predominance underlies SLE-accelerated AS (30).

The buildup of lipids, immune cells, and debris along with extracellular matrix (ECM) degradation and rising inflammation promotes unstable plaque development (31). vascular smooth muscle cell (VSMC)-mediated fibrous cap formation stabilizes plaques through ECM reinforcement (32). Neutrophil-induced endothelial injury disrupts this process, increasing thrombosis risk (33). Chronic SLE inflammation synergizes with traditional risk factors to create self-perpetuating cycles of plaque expansion/rupture, culminating in myocardial infarction or stroke (31). Therefore, targeting the oxidative stress-macrophage polarization axis may break this vicious cycle in SLE-associated AS.

### 2.2 Type I IFN response

**Figure 2:**
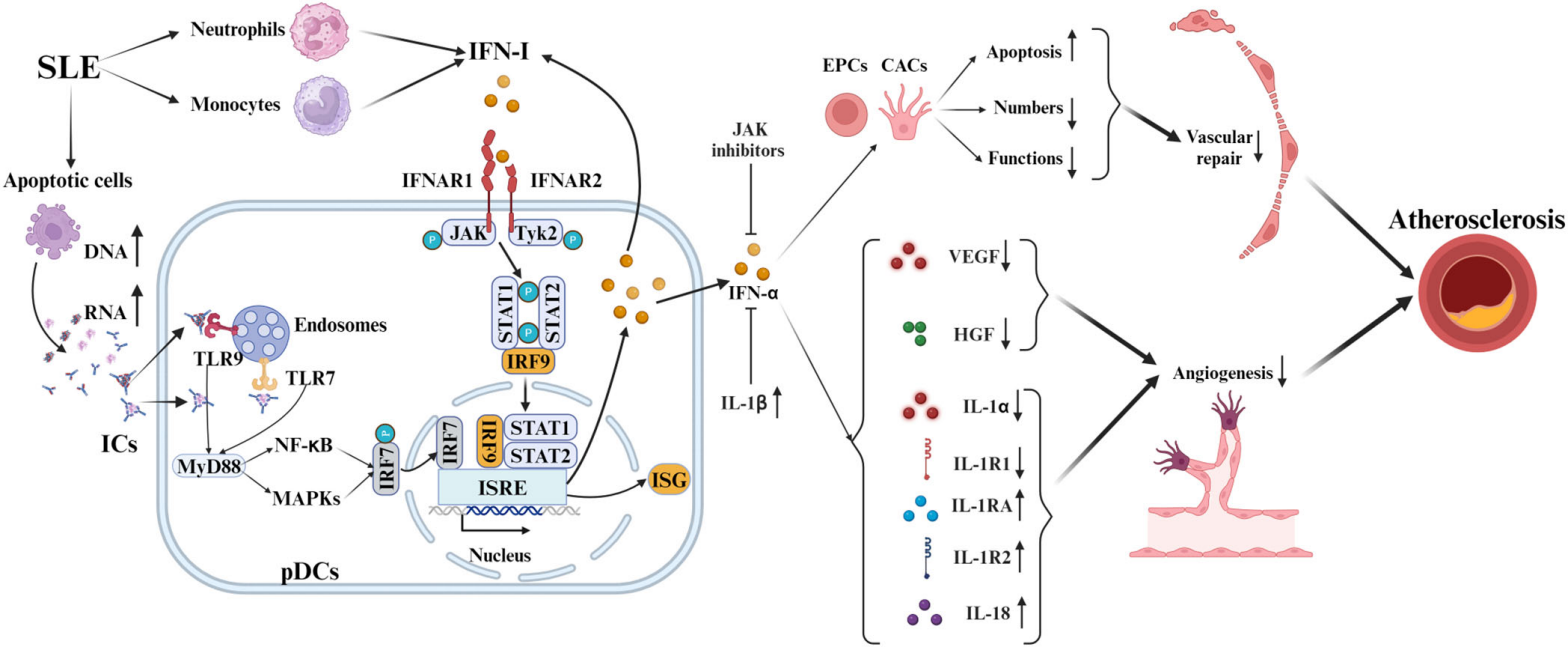
Mechanisms of IFN-I in SLE-associated atherosclerosis. In SLE, pDCs produce IFN-I via TLR7/9 sensing of immune complex nucleic acids, activating IRF7 and increasing IFN-I. IFN-I binding to IFNAR triggers JAK-STAT, enhancing ISG expression via IRF9. It causes endothelial injury and curbs angiogenesis by inhibiting VEGF and HGF, and modulates cytokines like IL-1 and IL-18, advancing SLE pathology. IFN-α also promotes EPC and CAC apoptosis and dysfunction. Counteracting IFN-α’s anti-angiogenic effects involves JAK inhibitors and IL-1β.

Type I interferons (IFN-I) are crucial in the development of SLE-Related AS, as depicted in **Figure 2**. In SLE, IFN-I production originates from dendritic cells, neutrophils, and monocytes/macrophages (21), encompassing thirteen IFN-α subtypes, IFN-β, and rare variants (IFN-ϵ, IFN-κ, IFN-ω) (34). Over 60% of SLE patients demonstrate an “IFN signature” characterized by elevated IFN-regulated gene activity, with nearly 50% exhibiting sustained high IFN-I levels linked to genetic predisposition and enhanced disease risk (35). Defective apoptosis in SLE increases extracellular DNA/RNA that complexes with anti-DNA/RNA antibodies to form immune complexes (ICs) (36). ICs activate plasmacytoid dendritic cells (pDCs), which are primary IFN-I producers in SLE (37). pDCs recognize DNA and RNA in immune complexes via TLR9 and TLR7, triggering myeloid differentiation factor 88 (MyD88) and downstream signaling through nuclear factor kappa B (NF-κB) and Mitogen-activated protein kinases (MAPKs) (37). Subsequent nuclear translocation of interferon regulatory factor 7 (IRF7) triggers IFN-I transcription via binding to interferon-stimulated response element (ISRE) promoters (37, 38). Autocrine amplification occurs through IFNAR-mediated JAK kinase (TYK2/JAK1) activation, inducing STAT1/2 phosphorylation and ISGF3 complex formation that enhances interferon-stimulated genes (ISGs) expression (21).

IFN-I-induced endothelial cell damage and repair-imbalance accelerate atherosclerosis (39). In SLE, IFN-α significantly exacerbates atherosclerosis by modulating endothelial progenitor cells (EPCs) and bone marrow-derived circulating angiogenic cells (CACs) (40). This modulation includes inducing EPC apoptosis, which negatively impacts their number, structure, and function, thus impairing vascular repair (41). Furthermore, IFN-α inhibits vascular endothelial growth factor (VEGF) and hepatocyte growth factor (HGF) activities, hindering neovascularization and repair (39). Inhibiting the IFNAR reverses EPC/CAC dysfunction in SLE, restoring angiogenic potential (39). In NZM-2328 mice, IFN-α worsens reduced endothelial function and EPC/CAC levels, which IFNAR knockout prevents (42).

Concurrently, IFN-α creates anti-angiogenic microenvironments via IL-1 pathway dysregulation (reduced IL-1α/1β/1R1; elevated IL-1Ra/1R2) that JAK inhibitors effectively counteract (40). IFN-I further induces pro-atherogenic cytokines like IL-18, exacerbating vascular injury and AS progression in SLE (43). In conclusion, Type I interferons play a crucial role in SLE, influencing immune responses and vascular damage.

### 2.3 Neutrophils and neutrophil extracellular traps

**Figure 3:**
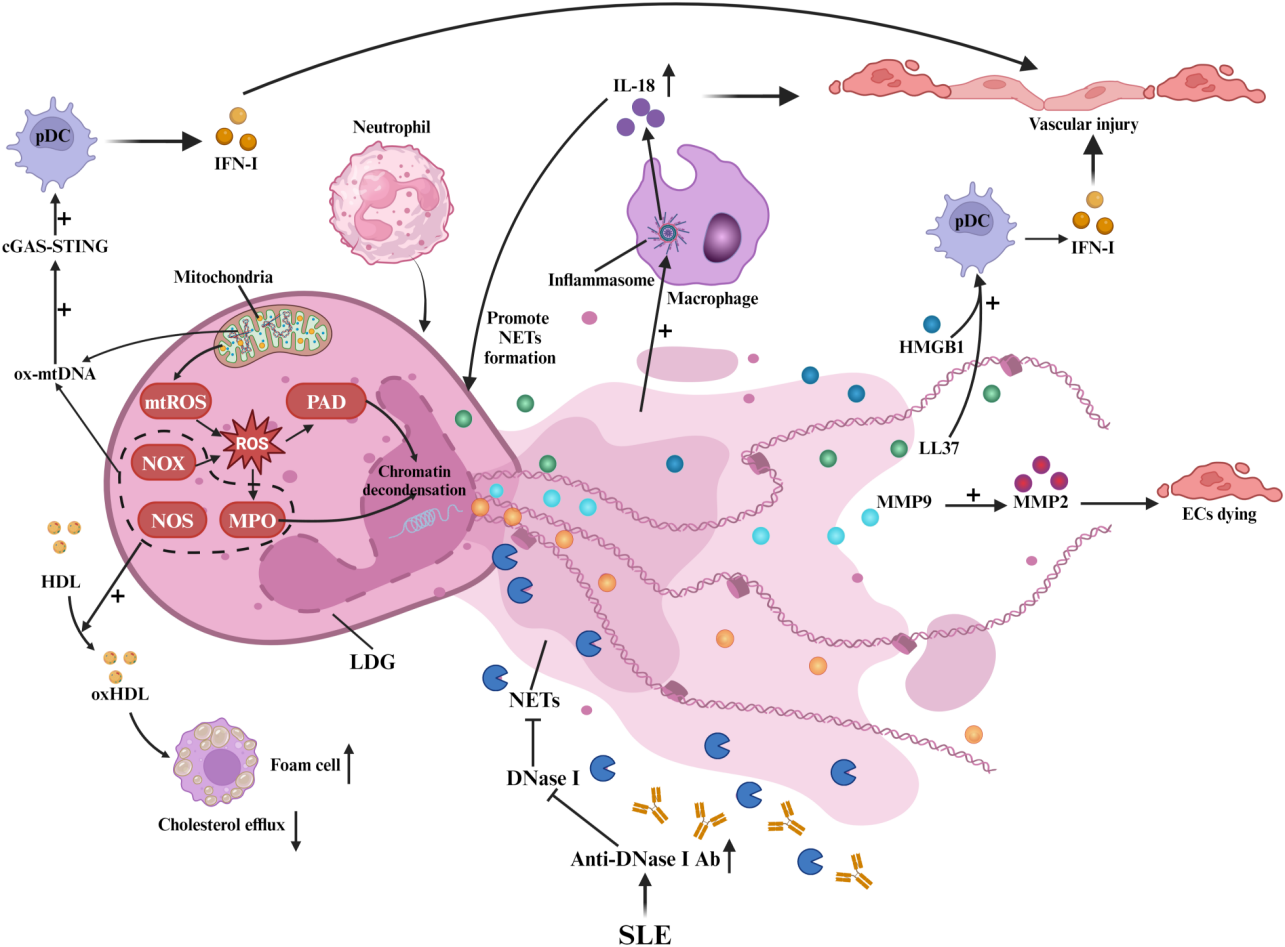
Neutrophils and NETs in SLE-associated atherosclerosis: Neutrophils and NETs. In SLE, LDGs release NETs, damaging ECs and triggering inflammation. NET formation involves ROS from NOX and mitochondria, with PAD and MPO inducing chromatin decondensation. Ox mtDNA activates cGAS-STING in pDCs, boosting IFN-I. NETs’ MMP9 activates MMP2, causing EC death. LL-37 and HMGB1 enhance pDC IFN-α, while inflammasome activity promotes IL-18. NOX, NOS, and MPO in NETs oxidize HDL, impairing cholesterol efflux. Anti-DNase I antibodies hinder NET clearance.

Neutrophils play a key role in SLE-related AS development by enhancing the IFN-I signature and NET formation, as shown in **Figure 3**. NETs are characterized by large, web-like structures composed of chromatin, histones, and proteins (44). The formation of these structures depends on ROS produced by NOX and/or mitochondrial ROS. Additionally, the activation of peptidyl arginine deiminase (PAD) and MPO converts arginine to citrulline, leading to chromatin decondensation (44).

Low-density granulocytes (LDGs), a pro-inflammatory neutrophil subset, drive spontaneous NET release that induces endothelial dysfunction and vascular injury in SLE-associated AS (45). These cells overproduce TNF-α and IFN-I (45), while their NETs deliver autoantigens that perpetuate autoimmune responses (46). LDG-derived NETs activate T-cells (47) and transfer matrix metalloproteinase 9 (MMP9) to endothelial cells, triggering MMP2-mediated apoptosis (48). NET-associated small RNAs promote endothelial inflammation through TLR signaling (47, 49), whereas LL-37 (a cathelicidin peptide) stabilizes NET-DNA complexes and enhances plasmacytoid dendritic cell (pDC)-mediated IFN-α production (4, 50). Concurrently, oxidized mitochondrial DNA (ox-mtDNA) in NETs activates the cyclic GMP-AMP synthase - stimulator of interferon genes (cGAS-STING) pathway, amplifying IFN-I responses (51, 52). Impaired NET clearance in SLE, attributed to anti-DNase I antibodies (53), allows persistent High Mobility Group Box 1 (HMGB1) release that exacerbates chemotaxis and cytokine storms (4, 54).

NETs further sustain vascular inflammation via macrophage inflammasome activation and IL-18-driven NETosis (55), establishing an IFN-I-NET positive feedback loop that accelerates AS (4, 5). NOX, nitric oxide synthase (NOS), and MPO within NETs oxidize HDL, impairing cholesterol efflux and promoting foam cell formation (56). Collectively, neutrophil-derived NETs emerge as central mediators of SLE-related AS through multifaceted immune activation and vascular injury mechanisms.

### 2.4 T-cell dysfunction

**Figure 4:**
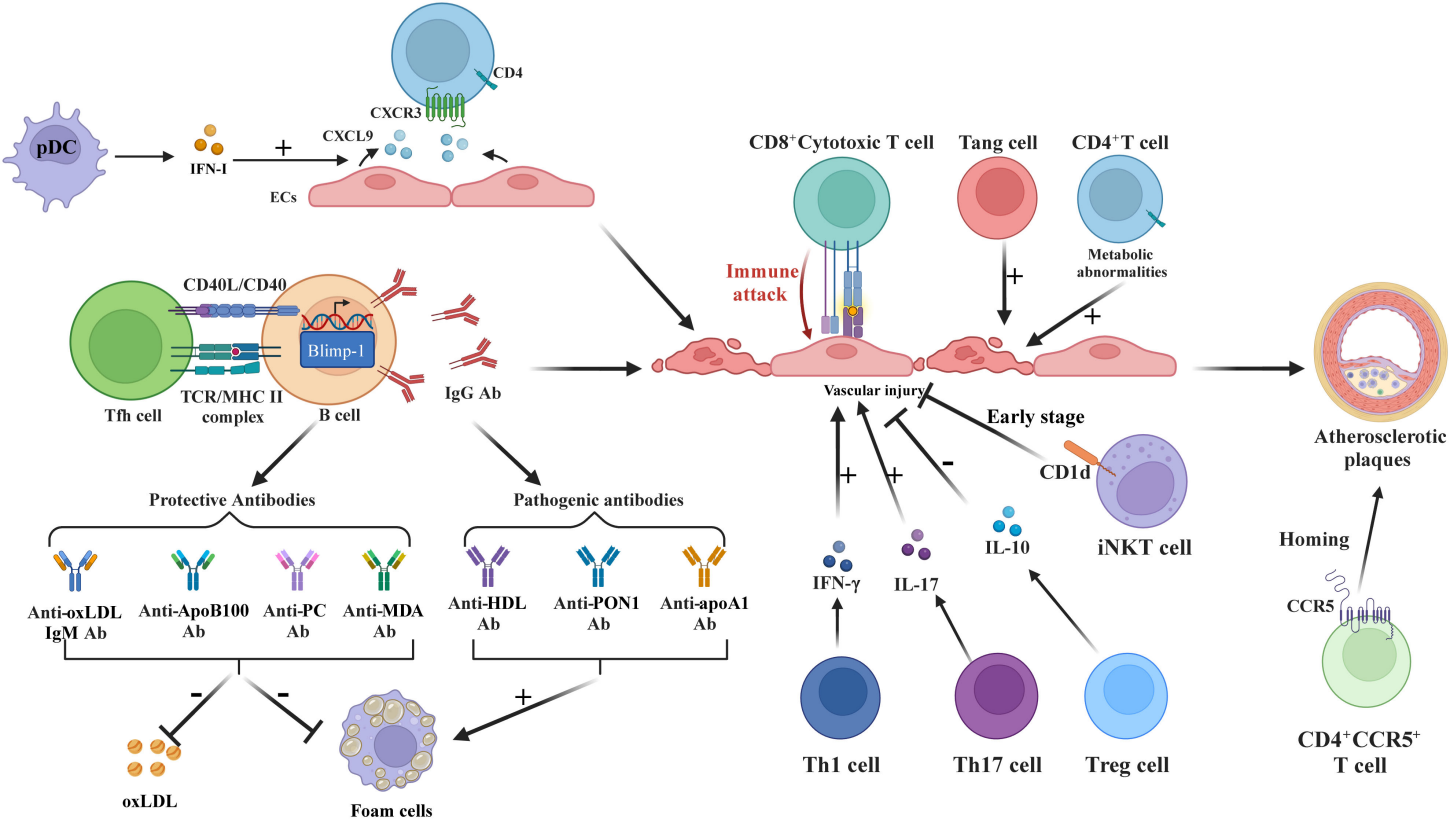
T and B Cells in SLE-associated atherosclerosis. IFN-I stimulates ECs to produce CXCR3 ligands, recruiting CD4^+^CXCR3^+^T-cells. Tfh enhances B-cell differentiation into IgG-secreting plasma cells. Th1/Th17 cells increase EC damage via IFN-γ and IL-17. CD8^+^T-cells disrupt ECs; Treg reduces inflammation via IL-10 and FoxP3. Tang cells aid repair but may worsen inflammation. iNKT initially protects but loses efficacy over time. CCR5 is essential for T-cell homing to atherosclerotic plaques. B-cells produce pathogenic antibodies (anti-HDL, anti-PON1, anti-apoA1) fostering foam cells, while protective antibodies (anti-oxLDL-IgM, anti-PC) lower oxLDL. SLE-related CD4^+^T-cell metabolic dysfunction exacerbates inflammation.

T-cell dysfunction is essential in the development of AS in SLE patients, as shown in **Figure 4**. In SLE, CD4^+^ T-cells exhibit hyperactivation with reduced activation thresholds (57), where pDC-derived IFN-I induces endothelial cell production of CXCR3 ligands (CXCL9), promoting CD4^+^CXCR3^+^ T-cell arterial infiltration (58). Additionally, CD4^+^CCR5^+^ T-cells accumulate in SLE patients with carotid AS, demonstrating CCR5-mediated plaque homing (59). These CXCR3^+^ (tissue migration) and CCR5^+^ (plaque recruitment) subsets synergistically accelerate AS through complementary pathways (60, 61), independent of traditional risk factors like dyslipidemia (62).

Follicular helper T-cells (Tfh) exacerbate AS via major histocompatibility complex II (MHCII)-dependent B-cell differentiation into IgG-secreting plasma cells (63, 64), while cTfh1/cTfh2/cTfh17 subpopulations interact with neutrophils to amplify vascular inflammation (65). Cholesterol-rich environments enhance splenic B-cell control over Tfh expansion through programmed death ligand 1 (PD-L1) regulation (63).

Imbalanced regulatory T-cell (Treg)/effector T-cell (Teff) ratios correlate with AS progression (62, 66). Dyslipidemia and elevated T-cell Pbx1d levels can intensify autoimmune reactions and atherosclerosis by disrupting Treg stability (62). Dyslipidemia-induced Pbx1d overexpression destabilizes Tregs, diminishing IL-10 and FoxP3 expression (66). Conversely, expanded Th17 cells secrete IL-17 to enhance plaque inflammation (66, 67), while Th1 and cytotoxic CD8+ T-cells directly damage endothelium via cytokine release (21, 68). Circulating IFN-I^hi^ CD4^+^ and exhausted/activated CD8^+^ T-cells infiltrate SLE vascular lesions (21, 69).

Other T-cell subsets, such as CD3^+^CD31^+^CXCR4^+^angiogenic T-cells (Tang), aid vascular endothelium repair during AS (70). However, in SLE patients, especially those positive for anti-dsDNA, elevated CD8^+^Tang cell levels in blood may signal endothelial damage and cardiovascular risks (71). The expanded CD28(null)Tang subset, associated with aging, intensifies endothelial inflammation via inflammatory factor release, leading to atherosclerosis and linked to SLE disease activity (72, 73). Invariant natural killer T (iNKT) cells lose early anti-AS capacity due to impaired CD1d-mediated lipid presentation (74, 75).

Metabolically reprogrammed CD4^+^ T-cells exhibit enhanced glycolysis via pyruvate kinase M2 (PKM2) activation, releasing pro-inflammatory cytokines (76). IFN-I disrupts T-cell bioenergetics (77), creating self-perpetuating inflammatory loops that pharmacological inhibition can reverse (78). Collectively, T-cell-mediated immunity constitutes a central axis in SLE-associated AS pathogenesis.

### 2.5 B-cell dysfunction

B-cells and their autoantibodies are key in SLE-induced AS, as shown in **Figure 4**. Elevated anti- HDL, paraoxonase 1 (PON1), and apolipoprotein A1 (apoA1) antibodies serve as progression markers (79, 80), with anti-HDL predicting cardiovascular risk and anti-PON1 correlating with intimal thickening (80). Patients with SLE exhibit reduced IgM/IgG anti-phosphocholine (PC) levels inversely associated with AS severity (81–85), where IgM anti-PC mitigates disease activity while IgG anti-PC inhibits endothelial adhesion molecules (83–85). Hyperlipidemia-driven B-cell activation promotes oxLDL generation (86, 87), further amplified by ICs containing ribonucleoproteins and anti-Ro/La antibodies that enhance anti-oxLDL production (88). OxLDL-β2-glycoprotein I complexes stimulate foam cell formation and autoantibody responses (89).

B-cell-activating factor (BAFF) elevation correlates with subclinical AS (90), while B1 cells paradoxically exhibit both protective (autoimmunity suppression) and pathogenic (class-switched IgG production) roles (91, 92). Lupus-prone mice demonstrate marginal zone B cell-driven pathogenic antibody secretion (93) and abnormal plasma cell differentiation involving somatic hypermutation (94). Pathogenic autoantibodies include anti-dsDNA (endothelial dysfunction/thrombosis induction) (95, 96), anti-endothelial cell antibodies (IgA-mediated vascular injury) (97, 98), and antiphospholipid antibodies (plaque progression in 20-30% patients) (99).

Protective autoantibodies counterbalance these effects: anti-oxLDL-IgM, anti-ApoB100, anti-PC, and anti-malondialdehyde (MDA) antibodies suppress oxLDL uptake and foam cell formation (100, 101). Reduced anti-ApoB100 levels in SLE imply impaired AS protection (101), while anti-C1q antibodies promote M2 macrophage polarization (102). Anti-PC-IgM modulates Treg/Th17 equilibrium, synergizing with anti-MDA-IgM for cardiovascular protection (84, 103). Nevertheless, dietary/metabolic constraints limit their therapeutic potential in SLE (104). Collectively, B-cell-derived autoantibody networks constitute pivotal drivers of SLE-associated AS.

## 3 The role of gut microbiota dysbiosis and metabolic disorders in the pathogenesis of atherosclerosis

Gut microbiota dysbiosis and metabolites (e.g., TMAO) are well - known to promote atherosclerosis via inflammatory pathways and immune dysregulation in various diseases, although their direct role in SLE associated atherosclerosis remains unclear. There are numerous studies exploring the relationships between SLE and gut microbiota, or between atherosclerosis and gut microbiota, and also between metabolic disorders and both of them. However, research comprehensively integrating SLE, atherosclerosis, gut microbiota, and metabolic disorders remains unexplored. While Cabana-Puig et al. (105) demonstrated high-fat diet (HFD) exacerbates atherosclerosis via Ly6C^+^ monocyte pathways and *Lactobacillus* improves glomerulonephritis, these interventions were examined independently. Thus, this study does not establish causal relationships between gut microbiota and SLE-associated atherosclerosis. This underscores the critical need for integrated investigations examining microbiome-atherosclerosis interactions specifically in SLE contexts.

**Figure 5:**
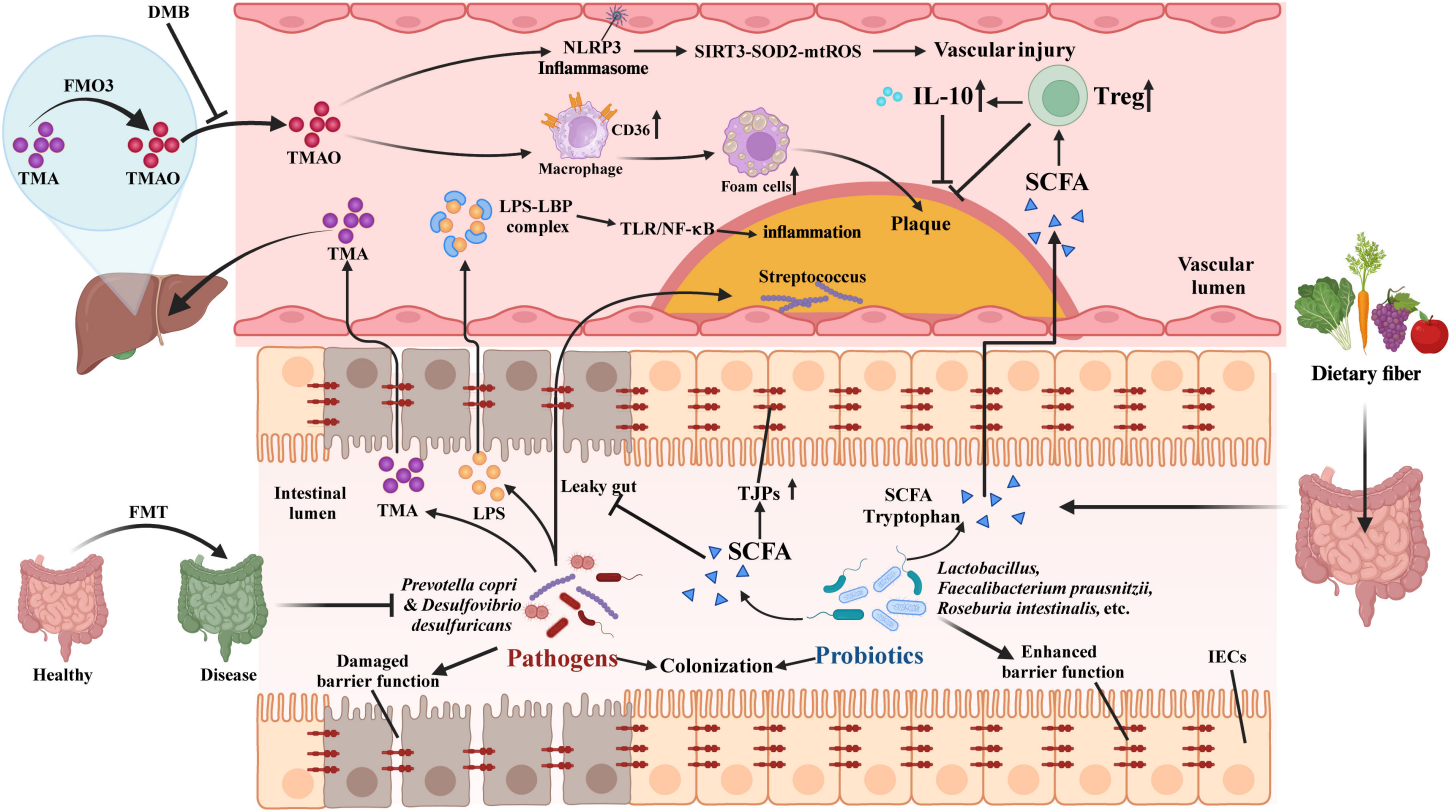
Potential mechanisms underlying gut microbiota dysbiosis in atherosclerosis. Pathogenic bacteria like *Prevotella copri* weaken the intestinal barrier, causing a “leaky gut” and allowing LPS into the bloodstream, which activates the LBP-TLR4/NF-κB pathway, triggering inflammation and promoting plaque formation. Streptococcus spp. can migrate to plaques, worsening the condition. Probiotics (e.g., *Lactobacillus*, *Faecalibacterium prausnitzii*) upregulate TJPs, reducing LPS-induced inflammation. Pathogenic bacteria produce TMA, converted to TMAO by FMO3, promoting atherosclerosis. DMB lowers TMAO and improves vascular function in lupus mice. SCFAs from probiotics enhance Tregs, and IL-10, and reverse cholesterol transport, preventing atherosclerosis. Probiotics, FMT, and dietary fiber modulate gut microbiota, lower TMA/TMAO and LPS, and reduce inflammation.

In this section, considering the current state of research, we mainly focus on the general mechanisms by which gut microbiota dysbiosis and metabolic disorders lead to atherosclerosis (including in diseases other than SLE), as depicted in **Figure 5**.

### 3.1 Genetic factors

The gut microbiota significantly influences the development of atherosclerosis, with genetic factors playing an important role. A study showed that proinflammatory gut microbiota from *Caspase1* ^-/-^ mice accelerated atherosclerosis in *Ldlr* ^-/-^ mice, as evidenced by increased levels of circulating monocytes, neutrophils, and proinflammatory cytokines. Fecal microbiota transplantation reduced short-chain fatty acid-producing taxa, such as *Akkermansia* and *Clostridium*, leading to decreased anti-inflammatory short-chain fatty acids in the cecum (9). This study highlights the multifaceted interplay between gut microbiota, genetic factors, and atherosclerosis.

### 3.2 Specific microbiota promotes atherosclerosis progression

Bacterial DNA in atherosclerotic plaques suggests intestinal bacteria can migrate to the vascular system, as seen with Streptococcus spp. moving to coronary artery plaques, leading to elevated pro-inflammatory cytokines and altered vascular tension, which contribute to coronary atherosclerosis (106, 107). Mechanistically, *Desulfovibrio desulfuricans* compromises intestinal barrier integrity, enabling lipopolysaccharide (LPS) translocation that activates the TLR4/NF-κB inflammatory axis (108). Circulating LPS subsequently recruits inflammatory cells to lesions through LPS-binding protein (LBP)/TLR4 signaling (109). Conversely, Faecalibacterium prausnitzii fortifies gut barriers and suppresses LPS synthesis, mitigating coronary artery disease (CAD) risk (110). Comparative studies reveal divergent microbial impacts: *Porphyromonas gingivalis* oral infection exacerbates atherosclerosis in *ApoE* ^-/-^ mice via gut dysbiosis and lipid abnormalities (reduced HDL/triglycerides) (111), while *Bacteroides fragilis*-induced dysbiosis (decreased *Lactobacillaceae*; increased *Desulfovibrionaceae*) promotes plaque formation through metabolic inflammation (10). Transplant experiments using *Caspase1* ^-/-^ mouse microbiota demonstrate accelerated atherosclerosis in *Ldlr* ^-/-^ recipients through systemic inflammation and microbial community shifts (9). These findings establish the gut-vascular axis as a multidimensional crosstalk involving barrier regulation, immunometabolic reprogramming, and microbial ecosystem remodeling.

### 3.3 TMAO as a promoter of atherosclerosis

TMAO accelerates atherosclerosis via multiple mechanisms. Gut microbes process dietary choline/carnitine/betaine into trimethylamine (TMA) using cut/cnt enzymes, with liver FMO3 converting TMA to TMAO (112). TMAO activates NF-κB to increase inflammatory cytokines for plaque formation (113), triggers NLRP3 inflammasome via SIRT3-SOD2-mtROS in endothelial cells (114), and enhances macrophage CD36/SR-A1-mediated ox-LDL uptake causing foam cell formation (115). It also suppresses bile acid synthesis through FXR/SHP receptors, impairing cholesterol excretion (116).

Gut microbiota directly modulates TMAO’s atherogenic effects. *Lachnoclostridium saccharolyticum* converts choline to TMA, correlating with plaque growth (117). *Emergencia timonensis* uniquely processes l-carnitine-derived γ-butyrobetaine into TMA (11). PSRC1 deficiency elevates TMAO and worsens atherosclerosis in *ApoE* ^-/-^ mice through gut dysbiosis (112).

Current research on TMAO in SLE remains limited. While TMAO’s pro-atherogenic mechanisms are well-characterized in other diseases (112–118), its specific role in SLE-related cardiovascular complications requires cautious interpretation given the limited disease-specific evidence. Clinical studies have demonstrated elevated plasma TMAO levels in lupus patients compared to healthy controls (119). González-Correa et al. (17) demonstrated that TMAO exacerbates vascular endothelial dysfunction via oxidative stress and impaired antioxidant defense in TLR7-activated lupus mice. Their intervention with 3,3-dimethyl-1-butanol (DMB), a TMA production inhibitor, effectively reduced circulating TMAO levels and alleviated hypertension in this lupus mouse model (17).

In conclusion, gut microbiota promotes TMAO production, which critically drives atherosclerosis through inflammatory and metabolic mechanisms (112–118). In lupus mice, TMAO causes vascular dysfunction that may accelerate atherosclerosis in SLE, but this connection requires further investigation. It should be emphasized that González-Correa et al. (17) specifically demonstrated TMAO’s contribution to vascular endothelial dysfunction in TLR7-activated SLE mice, but did not investigate atherosclerosis endpoints. To our knowledge, no published studies have directly examined TMAO’s effects on atherogenesis in lupus models or other systemic autoimmune diseases.

### 3.4 Tryptophan metabolism disruption

Recent studies have highlighted the role of tryptophan metabolism disruption in promoting atherosclerosis. A study showed that indoleamine 2,3-dioxygenase 1 (IDO1) promotes atherosclerosis by inhibiting IL-10 production, which activates a cAMP-dependent pathway and suppresses Erk1/2 phosphorylation, leading to increased plaque instability (120). Similarly, Laurans et al. found that IDO1 activity in obese mice disrupts the intestinal mucosal barrier, triggers chronic inflammation, and interferes with lipid metabolism, worsening obesity-related metabolic diseases (121). Additionally, Xue et al. identified a protective role for the gut microbial metabolite indole-3-propionic acid (IPA), which reduces plaque development by promoting reverse cholesterol transport in macrophages via the miR-142-5p/ABCA1 signaling pathway (122). These findings highlight the complex relationship between tryptophan metabolism and atherosclerosis.

### 3.5 Immune dysregulation

Recent research has highlighted the complex interactions between immune responses and gut microbiota in atherosclerosis development. One study found that under hyperlipidemic conditions, gut microbiota can activate B2 cells in perivascular adipose tissue, leading to increased IgG production and disease progression. Disrupting the gut microbiota with broad-spectrum antibiotics or depleting B2 cells pharmacologically mitigated atherosclerosis, emphasizing the role of B2 cells beyond lipid metabolism (123). Another study showed that immunization against gut bacterial outer membrane proteins reduced systemic inflammation and improved metabolic markers in mice fed a Western diet, mediated by apoE, suggesting a potential therapeutic approach for immune-metabolic diseases (124). Dietary modulation critically impacts this axis: high-fat/low-fiber diets induce microbial dysbiosis that amplifies mesenteric lymph node lymphocyte proliferation and accelerates plaque formation (125, 126).These findings underscore the importance of gut microbiota-immune interactions in atherosclerosis, offering insights into therapeutic strategies targeting the gut microbiome.

## 4 Therapeutic strategies for SLE-related atherosclerosis

### 4.1 Statins

Statins, cornerstone therapeutics for hyperlipidemia management, have garnered significant attention for their putative role in SLE-associated AS. Through HMG-CoA reductase inhibition, these agents inhibit cholesterol synthesis and ameliorate SLE-associated dyslipidemia—a pivotal atherosclerosis driver (5, 127). Preclinical studies demonstrate statins’ dual capacity to attenuate autoimmune activation and atherosclerotic progression (127), exerting anti-atherogenic effects through IL-6/IL-8/TNF-α suppression (5, 128). Clinical applications in SLE remain contentious (129): while observational data suggest cardiovascular risk reduction (130), the Lupus Atherosclerosis Prevention Study revealed no significant coronary artery or endothelial improvement after 24-month intervention (131). Current evidence thus indicates that despite plausible mechanistic benefits, statins’ clinical efficacy in SLE-related AS lacks robust validation, necessitating mechanistic investigations to delineate disease-specific pharmacodynamics and optimize therapeutic strategies.

### 4.2 Glucocorticoids

Glucocorticoids, cornerstone therapeutics for SLE, suppress inflammation and modulate atherogenesis through cytokine reduction (132, 133). Low-dose regimens (e.g., dexamethasone) demonstrate plaque-stabilizing effects (132, 133). However, dose-dependent cardiovascular risks emerge with prolonged high-dose administration, paradoxically accelerating plaque formation (133, 134). This bidirectional relationship necessitates strict adherence to minimal effective dosing with timely tapering to optimize risk-benefit ratios. Notably, chronic high-dose exposure elevates cardiovascular mortality in long-term treated cohorts, demanding vigilant monitoring (134). In conclusion, glucocorticoids should be administered with caution in SLE treatment, alongside other therapies, to balance their anti-inflammatory effects with the need to reduce cardiovascular hazards.

### 4.3 Immunosuppressant

Mycophenolate mofetil (MMF) and cyclophosphamide (CPA) demonstrate dual therapeutic potential in SLE-atherosclerosis pathogenic nexus. Preclinical evidence reveals MMF alleviates SLE manifestations and atherogenesis via inosine monophosphate dehydrogenase inhibition, curtailing lymphocyte-mediated inflammation (135). CPA exerts immunomodulatory effects through DNA synthesis disruption and lymphocyte clonal expansion blockade, synergistically attenuating plaque development (136). Clinical observations indicate MMF improves cardiovascular risk profiles in SLE cohorts (137, 138), though disease-specific clinical validation remains pending (139). While these immunosuppressants show mechanistic promise in atherosclerotic lesion regression, translating preclinical efficacy into optimized human SLE therapeutics requires systematic investigation of tissue-specific pharmacodynamics and long-term vascular outcomes.

### 4.4 Antimalarial drugs

The antimalarial agent hydroxychloroquine (HCQ) serves as a cornerstone therapy for SLE and its atherosclerotic complications. HCQ modulates SLE-associated atherogenesis through AP-1-mediated T-cell suppression (140) and endothelial stabilization via dual mechanisms: attenuation of TLR signaling and ROS generation, and AMPK-dependent VSMC cycle arrest (140, 141). Intriguingly, HCQ synergizes with high-dose statins to inhibit cholesterol biosynthesis through precursor regulation without altering serum cholesterol profiles (142). Multicenter clinical data validate its cardioprotective efficacy, demonstrating reductions in cholesterol levels, thrombotic events, and mortality rates in SLE cohorts (143). Extended analyses reveal 30% lower cardiovascular disease risk with HCQ use, particularly regarding CAD and cerebrovascular events (144, 145). These findings position HCQ as a dual-purpose therapeutic agent that concurrently addresses autoimmune dysregulation and vascular pathology in SLE management.

### 4.5 Therapeutic potential of interferon and NET inhibitors

IFN-I and NETs play a significant role in SLE-related AS, making them potential therapeutic targets. Anifrolumab, a monoclonal antibody, effectively treats moderate to severe SLE by blocking the type I interferon receptor, reducing NET formation, and lowering inflammatory cytokines (146). PADs—key enzymatic drivers of NET formation—show therapeutic promise by downregulating IFN-I signaling cascades in SLE-related AS (147). Janus kinase inhibitors (JAKi) including tofacitinib and baricitinib modulate SLE pathogenesis via JAK/STAT pathway inhibition, with preclinical evidence revealing tofacitinib’s dual capacity to reduce NET burden and restore endothelial homeostasis in MRL/lpr models (148–150). Early-phase clinical trials validate JAKi safety profiles and cardiometabolic benefits (149), while ruxolitinib extends this therapeutic spectrum in murine lupus (148). The TYK2 allosteric inhibitor deucravacitinib achieves comparable IFN-I suppression, with Phase II trials confirming both safety and clinical improvement in SLE cohorts (14, 21). Collectively, IFN-α antibodies and signaling inhibitors represent mechanistically grounded strategies for SLE-associated AS management, though definitive clinical validation requires large-scale trials to establish risk-benefit ratios across diverse patient populations.

### 4.6 Targeted T-cell therapy

The abnormal activation of T-cells plays an important role in atherosclerosis associated with SLE (67). For patients with SLE, Th17 cells exacerbate atherosclerosis advancement by generating inflammatory substances like IL-17 (66). Simultaneously, Tregs slow down atherosclerosis development by inhibiting effector T-cells and fostering anti-inflammatory reactions (151).

Notably, low-dose IL-2 selectively modulates the balance among Treg, Tfh, and Th17 cells, effectively reducing SLE disease activity and improving clinical outcomes while showing high tolerability and safety (15, 152). Consequently, a reduced dosage of IL-2 could decelerate atherosclerosis by controlling T-cells. To sum up, specific T-cell treatment methods may hold promise for atherosclerosis linked to SLE, yet additional studies are required to confirm their effectiveness and safety.

### 4.7 Targeted B cell therapy

B-cell-directed therapies emerge as viable interventions for SLE-linked AS. BAFF serves as a crucial modulator of B-cell homeostasis and SLE pathogenesis (153). The BAFF-neutralizing monoclonal antibody belimumab—an approved SLE biologic—demonstrates dual therapeutic capacity by achieving disease activity control and renal protection in lupus nephritis (153). Its anti-atherogenic effects exhibit context-dependent effects contingent upon lipidomic profiles and B-cell functional states, underscoring the necessity for precision targeting strategies (154). Clinical data reveal belimumab’s concomitant HDL elevation and Systemic Lupus Erythematosus Disease Activity Index (SLEDAI) score reduction, suggesting synergistic metabolic-immune benefits in AS mitigation (155). Complementary approaches employ CD20-directed agents like rituximab that mediate pathogenic B-cell depletion while preserving protective autoantibody pools, concurrently suppressing pathogenic T-cell cross-activation to impede AS progression (16, 156). Notably, rituximab ameliorates dyslipidemia in refractory SLE cases and attenuates myocardial infarction risk through pleiotropic immunometabolic mechanisms (16, 157). These findings position B-cell modulation as a strategic axis in SLE-related AS management, though future investigations must delineate molecular stratification biomarkers and establish dynamic monitoring protocols to optimize risk-adapted therapeutic strategies.

### 4.8 Potential therapies of targeting gut microbiota and metabolism in treating SLE-related atherosclerosis

Atherosclerosis is a significant cardiovascular complication in patients with SLE. Targeting gut microbiota and metabolic disorders has emerged as a novel approach to treat SLE-related atherosclerosis. Despite limited preclinical evidence, this approach holds significant promise for modulating both autoimmunity and vascular inflammation. Potential therapies include gene intervention, probiotic therapy, fecal microbiota transplantation, TMAO and tryptophan metabolism regulation, SCFA therapy and pharmacological or nutritional therapies. Relevant studies are listed in **Supplementary Table 1**.

#### 4.8.1 Gene intervention

Gene intervention may improve gut microbiota dysbiosis and metabolic abnormalities to benefit atherosclerosis. *NOD1*/*NOD2*-deficient mice had less atherosclerosis than *Ldlr* ^-/-^ mice after a 12-week high-fat diet, due to gut microbiota changes, increased intestinal cholesterol and coprostanol levels correlating with more Eubacterium coprostanoligenes, and lower plasma lipid levels and reduced foam cell formation from upregulated *Abca1* and *Abcg1* in macrophages (158). PSRC1 deficiency in *ApoE* ^-/-^ mice accelerated atherosclerosis by boosting TMAO, increased TMA-producing bacteria and plasma betaine and TMAO levels. These mice also showed colonic inflammation linked to gut microbiota dysregulation and PSRC1 deficiency increased liver flavin monooxygenase 3 (FMO3) expression, while PSRC1 overexpression suppressed FMO3 (112). These results highlight gene intervention’s potential to modulate gut microbiota and metabolic pathways to combat atherosclerosis, though more clinical research is needed for human validation.

#### 4.8.2 Probiotic therapy

A population study and *in vivo* experiments using *ApoE* ^-/-^ mice revealed that *Faecalibacterium prausnitzii* presence is associated with the lowest CAD incidence. *Faecalibacterium prausnitzii* reduces intestinal LPS synthesis, enhances mechanical and mucosal barriers, decreases plasma LPS levels, and alleviates atherosclerosis in *ApoE* ^-/-^ mice (110). A human study and *in vivo* experiments showed *Bacteroides vulgatus* and *Bacteroides dorei* abundance is reduced in CAD patients, while administering these bacteria alleviates atherosclerotic lesion formation in mice and reduces gut microbiota LPS production (159). Another study demonstrated that *Roseburia intestinalis* interacts with dietary plant polysaccharides to improve gut metabolism, reduce systemic inflammation, and significantly alleviate atherosclerosis. Intestinal butyrate administration can also reduce endotoxemia and atherosclerosis development (160). The research investigated a high-fat diet and interventions with LGG or telmisartan on atherosclerosis in *ApoE* ^-/-^ mice, finding both LGG and telmisartan significantly reduced atherosclerotic plaque size and improved various biomarkers (161). However, further clinical research is needed to validate these therapeutic effects in human patients.

#### 4.8.3 Fecal microbiota transplantation

FMT may be an effective strategy for treating atherosclerosis by modulating the gut microbiota. One study showed that Z-Ligustilide significantly alleviates atherosclerosis in *ApoE* ^-/-^ mice by reconstructing gut microbiota and maintaining intestinal barrier integrity via cannabinoid receptor 2 activation. Fecal bacteria from Z-Ligustilide-treated mice induced similar beneficial effects, and 16S RNA gene sequencing revealed a significant increase in *Rikenella* abundance in both Z-Ligustilide-treated and FMT mice (162). Another study showed Empagliflozin alleviates atherosclerosis, increases fecal probiotics abundance, reduces inflammatory responses, and alters gut microbiota metabolism; FMT from empagliflozin-treated mice to control mice also reduced atherosclerosis and systemic inflammatory responses (163). Furthermore, research using a C1q/TNF-related protein 9 gene-deficient (CTRP9-KO) mouse model found transplanting gut microbiota from wild-type mice into CTRP9-KO mice alleviated the development of atherosclerosis (164). However, further clinical research is needed to validate the efficacy and safety of FMT in human patients.

#### 4.8.4 TMAO regulation therapy

Targeting TMAO biosynthesis emerges as a novel therapeutic frontier for SLE-associated atherosclerosis. Pharmacological inhibition of TMA lyase by 3,3-dimethyl-1-butanol (DMB) in imiquimod-induced lupus models demonstrates multi-organ protection through TMAO suppression, including endothelial function restoration and macrophage foam cell formation inhibition independent of cholesterol modulation (17).

Complementary studies reveal diverse TMAO modulation strategies: Puerarin exerts atheroprotection via *Prevotella copri* depletion and TMA precursor reduction, while berberine’s vitamin-mimetic metabolite dihydroberberine disrupts gut microbial TMA biosynthesis (165, 166). Dietary interventions further expand this paradigm – aged garlic oligosaccharides reshape gut ecology toward *Akkermansia*-dominant communities with concomitant TMAO reduction in hyperlipidemic *ApoE* ^-/-^ models, whereas hickory polyphenols attenuate atherosclerosis through hepatic FMO3 transcriptional downregulation in high-choline-fed C57BL/6J mice (167, 168). Further clinical trials are necessary to confirm the efficacy and safety of these interventions in human patients.

#### 4.8.5 Tryptophan metabolism regulation therapy

Recent studies highlight tryptophan metabolism’s potential therapeutic role in atherosclerosis. A clinical trial and *in vivo* experiment in *ApoE* ^-/-^ mice revealed that IPA was significantly downregulated in CAD patients, correlating with atherosclerotic cardiovascular disease (ASCVD) risk and severity. Dietary IPA supplementation in *ApoE* ^-/-^ mice alleviated plaque development via the miR-142-5p/ABCA1 pathway by promoting cholesterol efflux. This study first uncovered the link between gut microbiota-derived tryptophan metabolites and ASCVD, providing a potential target (122). Another study showed that Aucubin improves atherosclerosis in *ApoE* ^-/-^ mice by modulating gut microbiota, particularly by increasing indole-3-acrylic acid (IAA). IAA alleviates atherosclerosis by activating the Aryl hydrocarbon receptor (AhR) and inhibiting the TGF-β/Smad pathway (169). Similarly, Indole-3-carbinol (I3C) inhibits atherosclerosis in *ApoE* ^-/-^ mice. I3C improves lipid profile, enhances gut microbiota diversity, and increases Verrucomicrobia abundance. Integrated analysis shows that 1-methyladenosine is a key modulator of I3C’s protective effect (170). These findings indicate that modulating tryptophan metabolism and gut microbiota could be promising therapeutic strategies for atherosclerosis, but more large-scale studies are needed to confirm their efficacy and safety in humans.

#### 4.8.6 Short-chain fatty acids regulation therapy

Short-chain fatty acids (SCFAs) show potential in treating atherosclerosis. Propionate (PA) reduces intestinal cholesterol absorption and lowers plasma LDL cholesterol via immune mechanisms, increasing regulatory T cells and IL-10 while inhibiting Npc1l1 expression. Oral PA supplementation in humans significantly decreases cholesterol levels, offering a new therapeutic target (171). In *ApoE* ^-/-^ mice, *Roseburia intestinalis* interacts with dietary plant polysaccharides to improve gut metabolism and reduce atherosclerosis, revealing gut microbiota and diet interaction mechanisms (160). These studies suggest SCFAs’ role in combating atherosclerosis through gut microbiota and immune modulation, though human validation is still required.

#### 4.8.7 Pharmacological or nutritional therapies

Recent studies have illuminated the potential of various treatments in improving atherosclerosis via gut microbiota modulation. One study found gut microbiota affects atherosclerosis development by regulating vascular microRNA-204 expression, which impacts vascular endothelial function by targeting Sirtuin1 (172). Bicyclol improves high-fat diet-induced atherosclerosis in *ApoE* ^-/-^ mice by restoring gut health and alleviating endothelial activation, macrophage infiltration, and cholesterol ester accumulation (173). Hydroxyurea effectively mitigates atherosclerosis in *ApoE* ^-/-^ mice by modulating gut microbiota and cholesterol metabolism, reducing plaque and liver lipid accumulation (174). Palmatine significantly mitigates atherosclerosis by modulating gut microbiota and phenylalanine metabolism, reshaping gut microbiota composition and reducing harmful bacteria abundance (175). Peanut Skin Extract significantly mitigates atherosclerosis in *ApoE* ^-/-^ mice by regulating lipid metabolism, exerting anti-inflammatory effects, and altering gut microbiota composition (176). Ginsenosides protect *Ldlr* ^-/-^ mice from atherosclerosis by enhancing bile salt hydrolase activity and protecting the intestinal barrier (177). ALW-II-41–27 significantly alleviates atherosclerosis in *ApoE* ^-/-^ mice by reshaping gut microbiota and modulating bile acid metabolism, reducing plaques and increasing collagen and smooth muscle cell content (178). Disulfiram reduces atherosclerosis in *ApoE* ^-/-^ mice by inhibiting GsdmD, inducing autophagy, and modulating gut microbiota (179). Dietary fiber also benefits SLE mice by modulating gut microbiota and improving vascular and renal health (180). These findings highlight the potential of targeting gut microbiota as a therapeutic strategy for atherosclerosis, with further human validation needed.

## 5 Future perspectives

SLE, a complex autoimmune disorder, increases the risk of atherosclerosis. Despite recent progress, challenges remain. This review connects SLE with atherosclerosis through immune imbalance, gut microbiota disturbances, and metabolic differences, and suggests future research and treatment strategies.

The paper revisits atherosclerosis risk factors in SLE, including both traditional cardiovascular risks and unique SLE pathophysiology. Recent studies highlight immune cell dysfunction, particularly macrophage metabolism imbalance and the role of LNGs and CD4^+^T-cells under type I interferon conditions, leading to mitochondrial damage and oxidative stress, which promote atherosclerosis. Lipid metabolism irregularities, disease activity also accelerate the condition.

Current therapies including statins, corticosteroids, immunosuppressants, antimalarials, and targeted molecular agents aim to modulate lipid profiles, inflammatory responses, immune activation, and gut microbiota composition to mitigate cardiovascular risks. Future investigations should prioritize uncovering novel atherogenic mechanisms in SLE, particularly immune-metabolic interactions in specialized cell populations.

Study limitations include predominant reliance on animal models and *in vitro* systems that may not accurately reflect human SLE pathophysiology. Disease heterogeneity necessitates rigorously designed clinical studies. Research priorities should focus on developing personalized therapies, elucidating immune cell contributions to vascular pathology, investigating gut microbiota-metabolism crosstalk, and identifying predictive biomarkers. The underexplored relationship between specific gut microbial species/metabolites and oxidative atherogenesis in SLE warrants particular attention, as this may reveal novel therapeutic targets and diagnostic tools.

Advances in SLE-related atherosclerosis research could inform cardiovascular management across autoimmune disorders. Multicenter clinical trials, interdisciplinary collaborations, and data-sharing platforms are critical for accelerating progress. Although challenges remain, emerging technologies offer optimism for developing precision therapies that improve SLE patients’ cardiovascular outcomes and overall quality of life. This synthesis enhances understanding of SLE-associated vascular pathogenesis and provides translational insights applicable to broader autoimmune research.

## Glossary

apoA1: Apolipoprotein A1
LDGs: Low-Density Granulocytes
AS: atherosclerosis
LOX-1: Lectin-like oxidized low-density lipoprotein receptor-1
ASCVD: atherosclerotic cardiovascular disease
LPS: Lipopolysaccharide
BAFF: B-cell-activating factor
MAPKs: Mitogen-activated protein kinases
CACs: circumferential artery cells
MDA: malondialdehyde
CAD: coronary artery disease
MMF: Mycophenolate mofetil
cGAS-STING: cyclic GMP-AMP synthase - stimulator of interferon genes pathway
MMP: Matrix Metalloproteinase
CPA: cyclophosphamide
MPO: Myeloperoxidase
CVDs: cardiovascular diseases
MyD88: myeloid differentiation factor 88
DMB: 3,3-dimethyl-1-butanol
NETs: Neutrophil Extracellular Traps
DNase I: Deoxyribonuclease I
NF-κB: Nuclear Factor Kappa B
ECM: extracellular matrix
NOX: NADPH Oxidase
EPCs: endothelial progenitor cells
NOS: Nitric Oxide Synthase
FMO3: flavin monooxygenase 3
Ox mtDNA: Oxidized Mitochondrial DNA
FMT: Fecal Microbiota Transplantation
oxLDL: Oxidized Low-Density Lipoprotein
HCQ: hydroxychloroquine
PAD: Peptidylarginine Deiminase
HDL: High-Density Lipoprotein
PC: Phosphatidylcholine
HFD: high-fat diet
pDCs: Plasmacytoid Dendritic Cells
HGF: hepatocyte growth factor
PD-L1: programmed death ligand 1
HMGB1: High Mobility Group Box 1
PKM2: pyruvate kinase M2
IAA: indole-3-acrylic acid
PON1: Paraoxonase 1
I3C: Indole-3-carbinol
ROS: Reactive Oxygen Species
ICAM-1: Intercellular Adhesion Molecule-1
SCFAs: Short-Chain Fatty Acids
ICs: immune complexes
SLE: systemic lupus erythematosus
IDO1: indoleamine 2,3-dioxygenase 1
SR-A1: scavenger receptor A1
IFN-I: Type I interferons
Tang cells: CD3+CD31+CXCR4+ angiogenic T-cells
TYK2/JAK1: IFNAR-mediated JAK kinase
Tfh: Follicular helper T-cells
iNKT: Invariant Natural Killer T-cells
Th1: CXCR3+ T helper 1 cells
IPA: indole-3-propionic acid
TJPs: Tight Junction Proteins
IRF: Interferon regulatory factor
TLR: Toll-like receptors
ISGs: interferon-stimulated genes
TMA: Trimethylamine
ISRE: interferon-stimulated response element
TMAO: Trimethylamine-N-Oxide
JAKi: Janus kinase inhibitors
VCAM-1: Vascular Cell Adhesion Molecule-1
LBP: Lipopolysaccharide Binding Protein
VEGF: vascular endothelial growth factor
LDL: low-density lipoprotein
VSMC: vascular smooth muscle cell.
