# Supplementary material for: Systemic lupus erythematosus and atherosclerosis: immune pathways and the uncharted territory of gut microbiota and metabolism
Source: Front Immunol. 2025 Apr 28;16:1492726. doi: 10.3389/fimmu.2025.1492726 (PMC12067219; doi:10.3389/fimmu.2025.1492726)
Supplement: Supplementary file 1 [file Table1.docx]

**Supplementary Table 1. Potential Therapies of Targeting Gut Microbiota and Metabolism in Treating SLE-Related Atherosclerosis**

| Year | Patients or  Mouse models | Main results | Conclusion | References |
| --- | --- | --- | --- | --- |
| 2020 | *Ldlr*^-/-^*Nod1/2*^-/-^ mice | *Ldlr*^-/-^*Nod1/2*^-/-^ mice exhibited less atherosclerosis and reduced lipid deposition in plaques after a high-fat diet compared to *Ldlr*^-/-^ mice. These mice showed altered gut microbiota, characterized by an increased abundance of *Eubacterium coprostanoligenes*, higher intestinal cholesterol and coprostanol levels, and lower plasma lipid levels. Additionally, there was reduced foam cell formation due to increased expression of cholesterol efflux transporters in macrophages. | *NOD1/2* deficiency alleviates atherosclerosis in *Ldlr*^-/-^ mice by modulating gut microbiota and cholesterol metabolism. | (1) |
| 2022 | *ApoE*^-/-^ mice | PSRC1 deficiency in *ApoE*^-/-^ mice accelerated plaque formation, increased TMA-producing bacteria, leading to higher plasma betaine and TMAO levels, and caused colonic inflammation associated with gut microbiota dysregulation. Additionally, PSRC1 deficiency increased liver FMO3 expression, while PSRC1 overexpression inhibited FMO3. | PSRC1 deficiency accelerates atherosclerosis by increasing TMAO production and gut microbiota dysregulation. | (2) |
| 2023 | CX3CR1-deficient MRL/lpr mice | CX3CR1-deficient MRL/lpr mice showed exacerbated glomerulonephritis versus wild-type, ameliorated via Lactobacillus-mediated microbiota modulation. HFD worsened atherosclerosis in these mice through Ly6C+ monocyte activation, which upregulated ICOS-L to engage ICOS+ Tfh cells, amplifying germinal center reactions and autoantibody production. | The study reveals new mechanisms of CX3CR1 deficiency leading to SLE-related glomerulonephritis and cardiovascular diseases, highlighting the important role of gut microbiota in disease development. | (3) |
| 2024 | *ApoE*^-/-^ mice | *Faecalibacterium prausnitzii* is associated with the lowest incidence of coronary artery disease among CAD groups and controls. *F. prausnitzii* reduces intestinal LPS synthesis and enhances mechanical and mucosal barriers, which leads to decreased plasma LPS levels and alleviates atherosclerosis in *ApoE*^-/-^ mice. | *F. prausnitzii* may help prevent coronary artery disease by inhibiting atherosclerosis. | (4) |
| 2018 | Coronary artery disease patients; *ApoE*^-/-^ mice | *Bacteroides vulgatus* and *Bacteroides dorei* are reduced in patients with coronary artery disease. Administration of these bacteria alleviates atherosclerotic lesion formation in mice and reduces gut microbiota LPS production. | *Bacteroides vulgatus* and *Bacteroides dorei* alleviate atherosclerosis by reducing LPS production from the gut microbiota. | (5) |
| 2018 | *ApoE*^-/-^ mice | *Roseburia intestinalis* interacts with dietary plant polysaccharides to improve gut metabolism, reduce systemic inflammation, and significantly alleviate atherosclerosis. Intestinal administration of butyrate can reduce endotoxemia and atherosclerosis development. | Increasing the proportion of butyrate-producing bacteria may protect against atherosclerosis. | (6) |
| 2016 | *ApoE*^-/-^ mice | The study investigated the effects of a high-fat diet and interventions with *Lactobacillus rhamnosus* GG (LGG) or telmisartan on atherosclerosis in *ApoE*^-/-^ mice. Both LGG and telmisartan significantly reduced atherosclerotic plaque size and improved various biomarkers. | LGG and telmisartan can improve atherosclerosis induced by a high-fat diet in *ApoE*^-/-^ mice. | (7) |
| 2024 | *ApoE*^-/-^ mice | Z-Ligustilide (ZL) alleviates atherosclerosis by improving gut microbiota and intestinal barrier. ZL enhances intestinal barrier function by activating the cannabinoid receptor 2 (CB2R), and fecal bacteria from ZL-treated mice induce similar beneficial effects on atherosclerosis and the intestinal barrier. 16S RNA sequencing shows increased *Rikenella* in ZL-treated and ZL FMT mice. | ZL significantly alleviates atherosclerosis in *ApoE*^-/-^ mice by activating CB2R and relying on gut microbiota. | (8) |
| 2023 | *ApoE*^-/-^ mice | Empagliflozin alleviates atherosclerosis and increases fecal probiotics by reducing inflammatory responses and altering gut microbiota metabolism. FMT from empagliflozin-treated mice to controls reduces atherosclerosis and systemic inflammatory responses. | Empagliflozin alleviates atherosclerosis in *ApoE*^-/-^ mice by regulating gut microbiota and metabolites. | (9) |
| 2022 | C1q/TNF-related protein 9 gene-deficient (CTRP9-KO) mice | CTRP9 gene deficiency is linked to gut microbiota in atherosclerosis. FMT from wild-type (WT) to CTRP9-KO mice alleviates atherosclerosis development, indicating that FMT affects disease progression. | Restoring gut microbial balance through fecal microbiota transplantation may be an effective strategy for treating atherosclerosis. | (10) |
| 2015 | *ApoE*^-/-^ mice | 3,3-dimethyl-1-butanol (DMB) inhibits TMA formation from cultured microbes and specific microbial TMA lyases. DMB reduces TMAO levels in mice fed a high-choline or L-carnitine diet, inhibits choline diet-enhanced macrophage foam cell formation, and atherosclerotic lesion development in *ApoE*^−/−^mice without altering cholesterol levels. | DMB inhibits gut microbial TMA production, reducing TMAO levels and suppressing atherosclerotic lesion development in *ApoE*^−/−^ mice, offering a new strategy for cardiovascular disease treatment. | (11) |
| 2024 | Atherosclerosis patients; *ApoE*^-/-^ mice | Puerarin (PU) alleviates atherosclerosis by modulating gut microbiota, specifically reducing *Prevotella copri* abundance and TMA production. The therapeutic effect of PU in atherosclerosis patients is associated with decreased levels of *P. copri* and plasma TMAO. | Puerarin alleviates atherosclerosis by targeting *P. copri* and TMA production. | (12) |
| 2022 | Atherosclerosis patients; High-Fat Diet-fed SD rats and hamsters | Berberine alleviates atherosclerosis by reducing TMAO biosynthesis in the gut. Its metabolite, dihydroberberine, lowers TMAO production via a vitamin-like mechanism. Moreover, berberine treatment decreases plaque scores in atherosclerosis patients. | Berberine alleviates atherosclerosis by modulating gut microbiota and reducing TMAO levels. | (13) |
| 2025 | *ApoE*^-/-^ mice | Aged garlic oligosaccharides (AGOs) significantly alleviate atherosclerosis induced by a high-fat and high-cholesterol diet. AGOs reduce plasma TMAO levels and aortic lipid deposition. Additionally, AGOs increase fecal short-chain fatty acid levels, decrease the Firmicutes/Bacteroidetes ratio, and promote *Akkermansia* dominance. | AGOs alleviate atherosclerosis in *ApoE*^-/-^ mice by modulating gut microbiota and metabolites. | (14) |
| 2024 | High-Fat Diet-fed C57BL/6J mice | Hickory polyphenol extracts improve obesity, inflammation, and atherosclerosis induced by a high-fat and high-choline diet by altering gut microbiota composition and function, increasing microbial abundance. The abundance of the CutC gene in gut microbiota is significantly reduced, consistent with TMA production. Moreover, hickory polyphenol extracts decrease liver FMO3 expression, thereby reducing serum TMAO levels. The metabolomic analysis identifies polyphenols with potential inhibitory effects on CutC/FMO3 activities. | Hickory polyphenol extracts mitigate atherosclerosis induced by a high-fat diet through gut microbiota and the TMA-FMO3-TMAO pathway regulation. | (15) |
| 2022 | coronary artery disease patients; *ApoE*^-/-^ mice | Tryptophan metabolite indole-3-propionic acid (IPA) is significantly downregulated in coronary artery disease (CAD) patients, correlating with atherosclerotic cardiovascular disease (ASCVD) risk and severity. Dietary IPA supplementation in *ApoE*^-/-^ mice alleviates atherosclerotic plaque development. IPA promotes cholesterol efflux from macrophages to ApoA-I via a miR-142-5p/ABCA1 signaling pathway, facilitating macrophage reverse cholesterol transport. Reduced IPA production leads to miR-142-5p overexpression in macrophages and accelerated atherosclerosis progression. The miR-142-5p/ABCA1/reverse cholesterol transport axis is dysregulated in CAD patients, correlating with circulating IPA levels. | IPA regulates macrophage cholesterol metabolism through the miR-142-5p/ABCA1 signaling pathway, affecting atherosclerosis progression, and providing new ideas for ASCVD treatment. | (16) |
| 2024 | *ApoE*^-/-^ mice | Aucubin improves atherosclerosis by modulating gut microbiota, specifically increasing indole-3-acrylic acid (IAA) derived from *Lactobacillus*. IAA alleviates atherosclerosis by activating the AhR and inhibiting the TGF-β/Smad pathway. This mechanism highlights the role of gut microbiota-derived metabolites in cardiovascular health. | Aucubin alleviates atherosclerosis in *ApoE*^-/-^ mice by modulating gut microbiota and metabolites, particularly by increasing IAA. | (17) |
| 2024 | *ApoE*^-/-^ mice | Indole-3-carbinol (I3C) inhibits atherosclerosis formation induced by a high-choline diet. I3C improves the lipid profile, enhances gut microbiota diversity, and increases *Verrucomicrobia* abundance. Additionally, 1-methyladenosine is identified as a key modulator of I3C's protective effect against atherosclerosis in high-choline-induced *ApoE*^-/-^ mice. | I3C alleviates atherosclerosis induced by a high-choline diet in *ApoE*^-/-^ mice through remodeling of the gut microbiome and metabolomics. | (18) |
| 2022 | *Ldlr*^-/-^ mice | Ginkgo biloba leaf extract (GbE) alleviates atherosclerosis induced by a high-fat diet by reshaping gut microbiota. Specifically, GbE decreases the Firmicutes/Bacteroidetes ratio while increasing the abundance of *Akkermansia*, *Alloprevotella*, *Alistipes*, and *Parabacteroides*. Moreover, GbE enhances gut microbiota metabolic functions, promoting the production of short-chain fatty acids, indole-3-acetate, and secondary bile acids. | GbE alleviates atherosclerosis in *Ldlr*^-/-^ mice by modulating gut microbiota and metabolites. | (19) |
| 2016 | *eSirt* ^-/-^ mice | Gut microbiota influences atherosclerosis development by regulating vascular microRNA-204 expression. MicroRNA-204 affects vascular endothelial function by targeting Sirtuin1 (Sirt1). | Gut microbiota affects atherosclerosis through the regulation of vascular microRNA-204 and Sirt1. | (20) |
| 2022 | *ApoE*^-/-^ mice | Bicyclol (BIC) ameliorates high-fat diet-induced atherosclerosis via gut microbiota regulation. BIC restores gut health, thereby improving systemic immune cell dynamics and liver function. Additionally, BIC alleviates endothelial activation, macrophage infiltration, and cholesterol ester accumulation, thus reducing plaque formation. | BIC mitigates atherosclerosis in *ApoE*^-/-^ mice by regulating gut microbiota and improving gut health. | (21) |
| 2022 | *ApoE*^-/-^ mice | Hydroxyurea mitigates atherosclerosis through modulation of gut microbiota and cholesterol metabolism. In *ApoE*^-/-^ mice fed a high-fat diet, it reduces plaque formation and liver lipid accumulation. Moreover, hydroxyurea alters gut microbiota composition, increasing the abundance of beneficial bacteria. | Hydroxyurea mitigates atherosclerosis in *ApoE*^-/-^ mice by regulating gut microbiota and cholesterol metabolism. | (22) |
| 2024 | *ApoE*^-/-^ mice | Palmatine (PAL) mitigates atherosclerosis by modulating gut microbiota and phenylalanine metabolism. PAL reduces plaque area and necrotic core, improving inflammatory infiltration. It reshapes gut microbiota composition, reducing *Desulfovibrio piger* (*D. piger*) abundance and serum hippuric acid (HA) level. *D. piger* converts phenylalanine into 3-phenylpropionic acid, a precursor of HA, influencing atherosclerosis development. | PAL mitigates atherosclerosis in *ApoE*^-/-^ mice by regulating gut microbiota and phenylalanine metabolism. | (23) |
| 2022 | *ApoE*^-/-^ mice | Peanut Skin Extract (PSE) mitigates high-fat diet-induced atherosclerosis by regulating lipid metabolism and exerting anti-inflammatory effects. Additionally, PSE alters gut microbiota composition, increasing the abundance of beneficial bacteria while reducing that of harmful bacteria. | PSE mitigates atherosclerosis in *ApoE*^-/-^ mice by regulating gut microbiota and exerting anti-inflammatory effects. | (24) |
| 2024 | *Ldlr*^-/-^ mice | Ginsenoside Rb1 increases *Lactobacillus* abundance, enhances bile salt hydrolase (BSH) activity, and promotes intestinal conjugated bile acids hydrolysis and excretion. It inhibits FXR-FGF15 signaling, increases *CYP7A1* transcriptional expression, and facilitates cholesterol metabolism and elimination. Ginsenoside Rg1 protects the intestinal barrier, reduces metabolic endotoxemia, induces mucin production, and maintains *Akkermansia muciniphila*, inhibiting intestinal permeability. These mechanisms contribute to the anti-atherosclerotic effects of ginsenosides, supported by studies on gut microbiota's role in cholesterol metabolism and atherosclerosis development. | Ginsenosides Rb1 and Rg1 protect *Ldlr*^-/-^ mice from atherosclerosis by regulating different signal nodes. | (25) |
| 2024 | *ApoE*^-/-^ mice | ALW-II-41-27 alleviates atherosclerosis by reshaping gut microbiota and modulating bile acid metabolism. It reduces plaques, increases collagen and smooth muscle cell content, lowers cholesterol levels, and reduces colonic inflammation. ALW-II-41-27 treatment enriches gut microbiota with beneficial bacteria, enhancing secondary bile acid production. Mice receiving feces from treated mice show reduced plaques. | ALW-II-41-27 significantly alleviates atherosclerosis in *ApoE*^-/-^ mice by reshaping gut microbiota and modulating bile acid metabolism. | (26) |
| 2024 | *ApoE*^-/-^ mice | Disulfiram reduces atherosclerosis by inhibiting GsdmD (Gasdermin D), inducing autophagy, and modulating atheroprotective pathways such as efferocytosis and phagocytosis. 16S rRNA sequencing reveals increased *Akkermansia* and decreased *Romboutsia* in Disulfiram-treated mice on a high-fat diet, indicating gut microbiota modulation. | Disulfiram alleviates atherosclerosis in high-fat diet-fed *ApoE*^-/-^ mice by modulating GsdmD-dependent and GsdmD-independent atheroprotective pathways. | (27) |

# REFERENCES

1. Vlacil AK, Schuett J, Ruppert V, Soufi M, Oberoi R, Shahin K *et al.*, Deficiency of Nucleotide-binding oligomerization domain-containing proteins (NOD) 1 and 2 reduces atherosclerosis. *Basic Res Cardiol* (2020) **115**: 47. doi: 10.1007/s00395-020-0806-2

2. Luo T, Guo Z, Liu D, Guo Z, Wu Q, Li Q *et al.*, Deficiency of PSRC1 accelerates atherosclerosis by increasing TMAO production via manipulating gut microbiota and flavin monooxygenase 3. *Gut Microbes* (2022) **14**: 2077602. doi: 10.1080/19490976.2022.2077602

3. Cabana-Puig X, Lu R, Geng S, Michaelis JS, Oakes V, Armstrong C *et al.*, CX(3)CR1 modulates SLE-associated glomerulonephritis and cardiovascular disease in MRL/lpr mice. *Inflamm Res* (2023) **72**: 1083-1097. doi: 10.1007/s00011-023-01731-1

4. Yang HT, Jiang ZH, Yang Y, Wu TT, Zheng YY, Ma YT *et al.*, Faecalibacterium prausnitzii as a potential Antiatherosclerotic microbe. *Cell Commun Signal* (2024) **22**: 54. doi: 10.1186/s12964-023-01464-y

5. Yoshida N, Emoto T, Yamashita T, Watanabe H, Hayashi T, Tabata T *et al.*, Bacteroides vulgatus and Bacteroides dorei Reduce Gut Microbial Lipopolysaccharide Production and Inhibit Atherosclerosis. *Circulation* (2018) **138**: 2486-2498. doi: 10.1161/CIRCULATIONAHA.118.033714

6. Kasahara K, Krautkramer KA, Org E, Romano KA, Kerby RL, Vivas EI *et al.*, Interactions between Roseburia intestinalis and diet modulate atherogenesis in a murine model. *Nat Microbiol* (2018) **3**: 1461-1471. doi: 10.1038/s41564-018-0272-x

7. Chan YK, Brar MS, Kirjavainen PV, Chen Y, Peng J, Li D *et al.*, High fat diet induced atherosclerosis is accompanied with low colonic bacterial diversity and altered abundances that correlates with plaque size, plasma A-FABP and cholesterol: a pilot study of high fat diet and its intervention with Lactobacillus rhamnosus GG (LGG) or telmisartan in ApoE(-/-) mice. *BMC Microbiol* (2016) **16**: 264. doi: 10.1186/s12866-016-0883-4

8. Liu SJ, Fu JJ, Liao ZY, Liu YX, He J, He LY *et al.*, Z-ligustilide alleviates atherosclerosis by reconstructing gut microbiota and sustaining gut barrier integrity through activation of cannabinoid receptor 2. *Phytomedicine* (2024) **135**: 156117. doi: 10.1016/j.phymed.2024.156117

9. Hao H, Li Z, Qiao SY, Qi Y, Xu XY, Si JY *et al.*, Empagliflozin ameliorates atherosclerosis via regulating the intestinal flora. *Atherosclerosis* (2023) **371**: 32-40. doi: 10.1016/j.atherosclerosis.2023.03.011

10. Kim ES, Yoon BH, Lee SM, Choi M, Kim EH, Lee BW *et al.*, Fecal microbiota transplantation ameliorates atherosclerosis in mice with C1q/TNF-related protein 9 genetic deficiency. *Exp Mol Med* (2022) **54**: 103-114. doi: 10.1038/s12276-022-00728-w

11. Wang Z, Roberts AB, Buffa JA, Levison BS, Zhu W, Org E *et al.*, Non-lethal Inhibition of Gut Microbial Trimethylamine Production for the Treatment of Atherosclerosis. *Cell* (2015) **163**: 1585-95. doi: 10.1016/j.cell.2015.11.055

12. Puerarin alleviates atherosclerosis via the inhibition of Prevotella copri and its trimethylamine production. *Gut* (2024) **73**: 1934-1943. doi: 10.1136/gutjnl-2024-331880

13. Ma SR, Tong Q, Lin Y, Pan LB, Fu J, Peng R *et al.*, Berberine treats atherosclerosis via a vitamine-like effect down-regulating Choline-TMA-TMAO production pathway in gut microbiota. *Signal Transduct Target Ther* (2022) **7**: 207. doi: 10.1038/s41392-022-01027-6

14. Wang X, Cui J, Gu Z, Guo L, Liu R, Guo Y *et al.*, Aged garlic oligosaccharides modulate host metabolism and gut microbiota to alleviate high-fat and high-cholesterol diet-induced atherosclerosis in ApoE(-/-) mice. *Food Chem* (2025) **463**: 141409. doi: 10.1016/j.foodchem.2024.141409

15. Jiang C, Wang S, Wang Y, Wang K, Huang C, Gao F *et al.*, Polyphenols from hickory nut reduce the occurrence of atherosclerosis in mice by improving intestinal microbiota and inhibiting trimethylamine N-oxide production. *Phytomedicine* (2024) **128**: 155349. doi: 10.1016/j.phymed.2024.155349

16. Xue H, Chen X, Yu C, Deng Y, Zhang Y, Chen S *et al.*, Gut Microbially Produced Indole-3-Propionic Acid Inhibits Atherosclerosis by Promoting Reverse Cholesterol Transport and Its Deficiency Is Causally Related to Atherosclerotic Cardiovascular Disease. *Circ Res* (2022) **131**: 404-420. doi: 10.1161/CIRCRESAHA.122.321253

17. Luo Z, Yang L, Zhu T, Fan F, Wang X, Liu Y *et al.*, Aucubin ameliorates atherosclerosis by modulating tryptophan metabolism and inhibiting endothelial-mesenchymal transitions via gut microbiota regulation. *Phytomedicine* (2024) **135**: 156122. doi: 10.1016/j.phymed.2024.156122

18. He Y, Zhu Y, Shui X, Huang Z, Li K, Lei W, Gut microbiome and metabolomic profiles reveal the antiatherosclerotic effect of indole-3-carbinol in high-choline-fed ApoE(-/-) mice. *Phytomedicine* (2024) **129**: 155621. doi: 10.1016/j.phymed.2024.155621

19. Wang Y, Xu Y, Xu X, Wang H, Wang D, Yan W *et al.*, Ginkgo biloba extract ameliorates atherosclerosis via rebalancing gut flora and microbial metabolism. *Phytother Res* (2022) **36**: 2463-2480. doi: 10.1002/ptr.7439

20. Vikram A, Kim YR, Kumar S, Li Q, Kassan M, Jacobs JS *et al.*, Vascular microRNA-204 is remotely governed by the microbiome and impairs endothelium-dependent vasorelaxation by downregulating Sirtuin1. *Nat Commun* (2016) **7**: 12565. doi: 10.1038/ncomms12565

21. Li XL, Cui JJ, Zheng WS, Zhang JL, Li R, Ma XL *et al.*, Bicyclol Alleviates Atherosclerosis by Manipulating Gut Microbiota. *Small* (2022) **18**: e2105021. doi: 10.1002/smll.202105021

22. Yang XY, Yu H, Fu J, Guo HH, Han P, Ma SR *et al.*, Hydroxyurea ameliorates atherosclerosis in ApoE(-/-) mice by potentially modulating Niemann-Pick C1-like 1 protein through the gut microbiota. *Theranostics* (2022) **12**: 7775-7787. doi: 10.7150/thno.76805

23. Wang A, Guan B, Yu L, Liu Q, Hou Y, Li Z *et al.*, Palmatine protects against atherosclerosis by gut microbiota and phenylalanine metabolism. *Pharmacol Res* (2024) **209**: 107413. doi: 10.1016/j.phrs.2024.107413

24. Xu M, Lv C, Wang H, Lu Q, Ye M, Zhu X *et al.*, Peanut skin extract ameliorates high-fat diet-induced atherosclerosis by regulating lipid metabolism, inflammation reaction and gut microbiota in ApoE(-/-) mice. *Food Res Int* (2022) **154**: 111014. doi: 10.1016/j.foodres.2022.111014

25. Wang Y, Wu J, Hong Y, Zhu J, Zhang Y, Zhang J *et al.*, Ginsenosides retard atherogenesis via remodelling host-microbiome metabolic homeostasis. *Br J Pharmacol* (2024) **181**: 1768-1792. doi: 10.1111/bph.16320

26. Lu C, Liu D, Wu Q, Zeng J, Xiong Y, Luo T, EphA2 blockage ALW-II-41-27 alleviates atherosclerosis by remodeling gut microbiota to regulate bile acid metabolism. *NPJ Biofilms Microbiomes* (2024) **10**: 108. doi: 10.1038/s41522-024-00585-7

27. Traughber CA, Timinski K, Prince A, Bhandari N, Neupane K, Khan MR *et al.*, Disulfiram Reduces Atherosclerosis and Enhances Efferocytosis, Autophagy, and Atheroprotective Gut Microbiota in Hyperlipidemic Mice. *J Am Heart Assoc* (2024) **13**: e033881. doi: 10.1161/JAHA.123.033881
